# Combinatorial drug screening identifies synergistic co-targeting of Bruton's tyrosine kinase and the proteasome in mantle cell lymphoma

**DOI:** 10.1038/leu.2013.249

**Published:** 2013-10-08

**Authors:** M Axelrod, Z Ou, L K Brett, L Zhang, E R Lopez, A T Tamayo, V Gordon, R J Ford, M E Williams, L V Pham, M J Weber, M L Wang

**Affiliations:** 1 Department of Microbiology, Immunology and Cancer Biology, University of Virginia Health System, Charlottesville, VA, USA; 2 Department of Lymphoma and Myeloma, University of Texas MD Anderson Cancer Center, Houston, TX, USA; 3 Division of Hematology/Oncology, University of Virginia Health System, Charlottesville, VA USA; 4 Department of Hematopathology, University of Texas MD Anderson Cancer Center, Houston, TX, USA

We have performed a focused combinatorial screen of targeted drugs combined with ibrutinib in mantle cell lymphoma (MCL) cells, and identified the proteasome inhibitor carfilzomib as a targeted agent that could be used with ibrutinib to provide improved clinical responses. Other targeted agents that displayed cytotoxic benefit in our screen also were independent of the B-cell receptor (BCR) pathway, whereas agents within the BCR pathway did not provide benefit.

MCL is an incurable B-cell malignancy with poor prognosis.^1, 2^ As with many other malignancies and lymphoproliferative disorders of B-cell lineage, growth and survival of MCL depends on signaling via the BCR.^3, 4^ Potential therapeutic targets of the BCR pathway for MCL include downstream kinases LYN, SYK, PI3K and Bruton's tyrosine kinase (BTK). Ibrutinib (PCI-32765) is an orally bioavailable BTK inhibitor, which has clinical efficacy against numerous B-cell malignancies. In phase I/II clinical trials, ibrutinib elicited an overall response rate of 68% in patients with relapsed/refractory MCL, including patients previously exposed to bortezomib^3^ and 83% in patients with relapsed/refractory chronic lymphocytic leukemia (CLL).^4^ This is the highest response rate demonstrated by any single agent in MCL and CLL.

However, in spite of these encouraging results, responses are generally incomplete, *de novo* resistance is common and recurrence is anticipated, as is the case with most single-agent targeted therapies.^5^ Treatment with a single-agent targeted drug rapidly activates a variety of redundant and compensatory signaling pathways that blunt cytotoxicity and rapidly lead to adaptive resistance.^5, 6^ Consequently, disease progression or recurrence can occur within months and is often more clinically aggressive and resistant to treatment than at initial presentation. Although the mechanisms of primary and acquired resistance to ibrutinib have yet to be elucidated, anecdotal reports suggest that MCL disease progression on ibrutinib can be aggressive and often refractory to other treatments, indicating that compensatory signaling changes and adaptive resistance have occurred. In addition, acquisition of mutations of BTK that impact ibrutinib binding was recently observed in CLL cells.^7^ We hypothesize that drug combinations that block adaptive signaling responses can elicit deeper and broader initial remissions and may enable prolongation of both progression-free and overall survival in MCL.^8, 9, 10^ Therefore, we have aimed to identify drugs to combine with ibrutinib that target these adaptive responses, and that may also provide benefit in cases of acquired BTK mutations.

We constructed a focused drug panel that contained agents with targets ‘inside' and ‘outside' the canonical BCR pathway, as defined by the KEGG database (Kyoto Encyclopedia of Genes and Genomes).^11^ Co-targeting inside the BCR pathway can provide insight into the potential for pathway reactivation as a mechanism of resistance; co-targeting outside the BCR pathway could reveal novel or unexpected functional relationships and synergies. Our results surprisingly showed no benefit from combining ibrutinib with drugs that targeted within the BCR pathway, but robust synergism between ibrutinib and several agents targeting outside the BCR pathway. In particular, we identified proteasome inhibitors, notably carfilzomib, that could provide an enhanced benefit to MCL patients and should be tested in a clinical setting.

We initially performed combinatorial screening of a pair of MCL cell lines using ibrutinib in pairwise combinations with a library of 14 other drugs and looked for synergistic cytotoxicity (see Supplementary Table 1 and Figure 1b). Using three doses of ibrutinib and secondary agents, we treated cells with a 3 × 3 combination matrix for 72 h (Figure 1a) and used the Bliss model of independence to score for synergy.^12^ Bendamustine, which targets outside the BCR pathway and is clinically effective in combination with ibrutinib,^13^ was used as a positive control. In agreement with prior reports, the combination of bendamustine and ibrutinib produced a robust synergistic cytotoxic response in both MCL cell lines tested (Figure 1b). This served as a validation for our screening and analytical methodologies.

Unsupervised hierarchical cluster analysis revealed that synergistic cytotoxicities occurred only with inhibitors that target outside the BCR pathway (Figure 1b). These results suggest that intrinsic and adaptive resistance to BTK inhibition by ibrutinib is not mediated by feedback reactivation of the BCR signaling pathway. By contrast, several agents inhibiting targets outside the BCR pathway conferred synergistic cytotoxicity in combination with ibrutinib. Specifically, high degrees of synergy and cytotoxicity were demonstrated with ibrutinib in combination with the proteasome inhibitors bortezomib or carfilzomib, or the BCL2 inhibitor ABT-199 (Figure 1c).

A recent report suggests that the combination of ibrutinib and bortezomib may be a viable therapeutic approach for MCL.^14^ Therefore, we further examined the synergistic cytotoxicities observed when combining ibrutinib with proteasome inhibitors. Carfilzomib, in combination with ibrutinib, resulted in robust synergy and cytotoxicity in a variety of cell lines and in primary samples from patients (Figure 2a). This effect was observed over a range of dose combinations in the cell lines used in the screen (Supplementary Figures 1A and B). A similar, but less robust, effect was observed with the combination of ibrutinib and bortezomib across the range of doses tested (Supplementary Figures 1C and D). Analysis of apoptosis revealed that the synergistic cytotoxicity could be accounted for by enhanced apoptosis (Figure 2b and Supplementary Figure 2). The combination of ibrutinib and carfilzomib regularly produced a greater apoptotic response than ibrutinib and bortezomib (data not shown).

To better assess the translational potential of this drug combination, we used a model in which *ex vivo* human MCL tumor samples are introduced into SCID-hu mice.^15^ We injected 5 × 10^6^ freshly isolated primary MCL cells into human fetal bone implanted in the SCID-hu mice. The level of circulating human β_2_M in mouse serum was used to monitor tumor burden in the SCID-hu mice. When human β_2_M was detected in mouse serum (data not shown), the mice received treatments with ibrutinib 25 mg/kg/day, oral gavage, daily and/or carfilzomib (CFZ) 5 mg/kg/day, IV, twice a week for 5 weeks. Overall, these data clearly demonstrate that both ibrutinib and CFZ as single agents, had anti-tumor effects *in vivo* (*P*<0.01, compared with vehicle control, Figure 2c). However, we found that ibrutinib in combination with carfilzomib increased survival by threefold in the primary MCL-bearing SCID-hu mice at 125 days compared with ibrutinib or CFZ alone (*P*<0.01, Figure 2c).

In summary, we screened pairwise drug combinations of ibrutinib with 14 other inhibitors that either targeted the BCR signaling pathway or are being actively tested/used in MCL and other B-cell malignancies. Our goal was to identify clinically actionable drug combinations in a relatively unbiased manner. We found that all of the drug combinations that produced robust synergy involved ibrutinib in combination with inhibitors of non-BCR pathway targets. Drugs inhibiting proximal targets directly, for example, dasatinib, enzastaurin, temsirolimus, R-788, SC-514 and idelalisib, did not confer synergistic cytotoxicity in combination with ibrutinib. Rather, these drugs sometimes antagonized the cytotoxic effect of ibrutinib alone. Although further investigation is needed, these data suggest that incomplete BCR pathway inhibition or pathway reactivation is not a common mechanism of adaptive ibrutinib resistance in MCL. Rather, a compensatory bypass of the BCR pathway by alternative signaling pathways could at least in some cases be responsible for resistance and progression of MCL. This strikingly contrasts with the mechanisms of adaptive resistance that appear in BRAF V600E melanomas, where adaptive resistance to vemurafenib occurs almost always via mechanisms that reactivate mitogen activated protein kinase signaling.^5^ The ability to uncover novel functional interactions between signaling pathways that appear unconnected is a major benefit of the drug screening approach.

In our studies, the amount of apoptosis detected upon treatment with the carfilzomib combination was significantly higher than that of the bortezomib combination. Carfilzomib, while highly active in relapsed multiple myeloma, is less well studied in MCL. However, carfilzomib's safety profile is superior to bortezomib's, with less reported neuropathy, making it a more attractive potential clinical agent for combination with ibrutinib.^16^ Therefore, we chose to focus on the combination of carfilzomib and ibrutinib, despite the current use of bortezomib in relapsed MCL. Carfilzomib targets the chymotrypsin-like protease more specifically than bortezomib does, and its higher degree of synergy when combined with ibrutinib compared with the bortezomib plus ibrutinib combination may be due to a variation in the specific mechanism of proteasome inhibition.^17^ All four cell lines responded to the combination of proteasome and BTK inhibition, including Jeko-1, a leukemic, classically indolent form of MCL, and Z138, a blastic, characteristically aggressive form of MCL, suggesting that the carfilzomib and ibrutinib combination may prove efficacious regardless of variations in specific patient MCL tumor biology. Interestingly, the carfilzomib and ibrutinib combination synergized even in the carfilzomib-resistant Rec-1 MCL cells, and in a patient sample that displayed carfilzomib resistance, implying that the combination restores efficacy of carfilzomib. The synergistic effect was seen in all *in vitro* MCL cell lines, as well as with *ex vivo* patient samples. This finding translated to a MCL-SCID-hu mouse model, where the combination improved survival at 125 days by threefold compared with either drug individually. Given the robust synergy demonstrated independently at our two institutions as well as the dramatic increase in overall survival in our mouse model, we have designed a phase I/II clinical trial with ibrutinib in combination with carfilzomib in relapsed/refractory MCL.

## Figures and Tables

**Figure 1:**
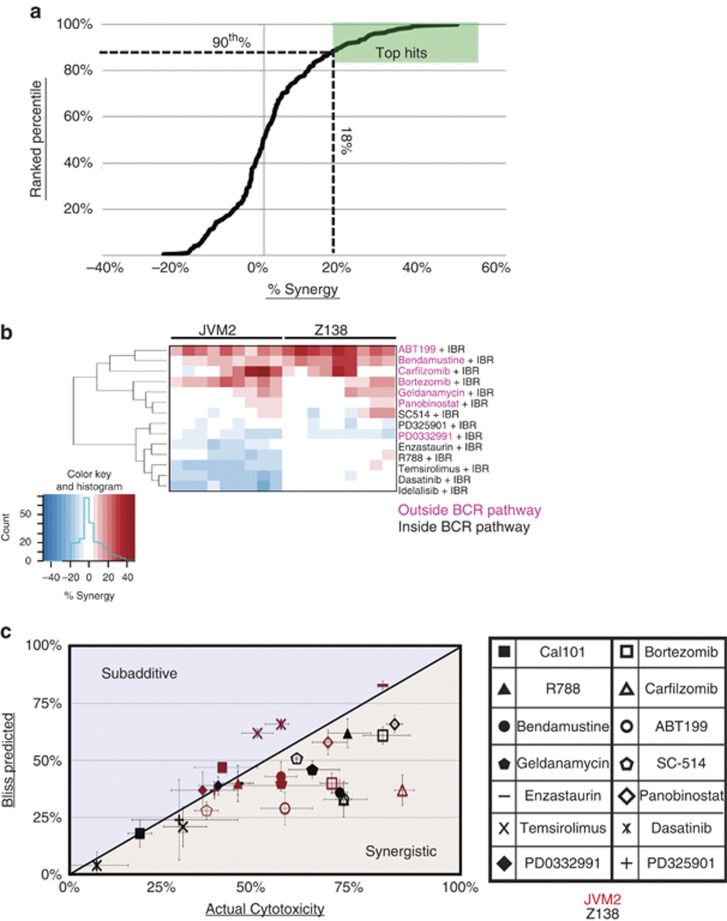
Screening with targeted secondary agents identifies drug combinations that synergize with ibrutinib. (**a**) Fourteen secondary drugs were combined with ibrutinib in two MCL cell lines (Z138 and JVM2). Cell lines were exposed to 6, 12 and 21 μM ibrutinib and three doses of secondary agents in a 3 × 3 format for 72 h (Supplementary Table 1). Percent cytotoxicity was measured with an alamarBlue assay, and percent synergy assessed by the Bliss independence method.^12^ Cytotoxicity was normalized to the vehicle-treated control samples for each cell line. Each data point on the curve represents the difference between the observed cytotoxicity and the predicted additive cytotoxicity based on the Bliss model (termed ‘percent synergy'). A cutoff was drawn at the 90th percentile, which corresponded to 18% synergy. (**b**) Unsupervised hierarchical clustering of the percent synergy values for all drug combinations at all concentration combinations in the 3 × 3 dosing matrix in Z138 and JVM2 cells. (**c**) Best synergistic response with associated percent cytotoxicity for all drug combinations in Z138 and JVM2 cells. Bendamustine was used as a positive control.

**Figure 2:**
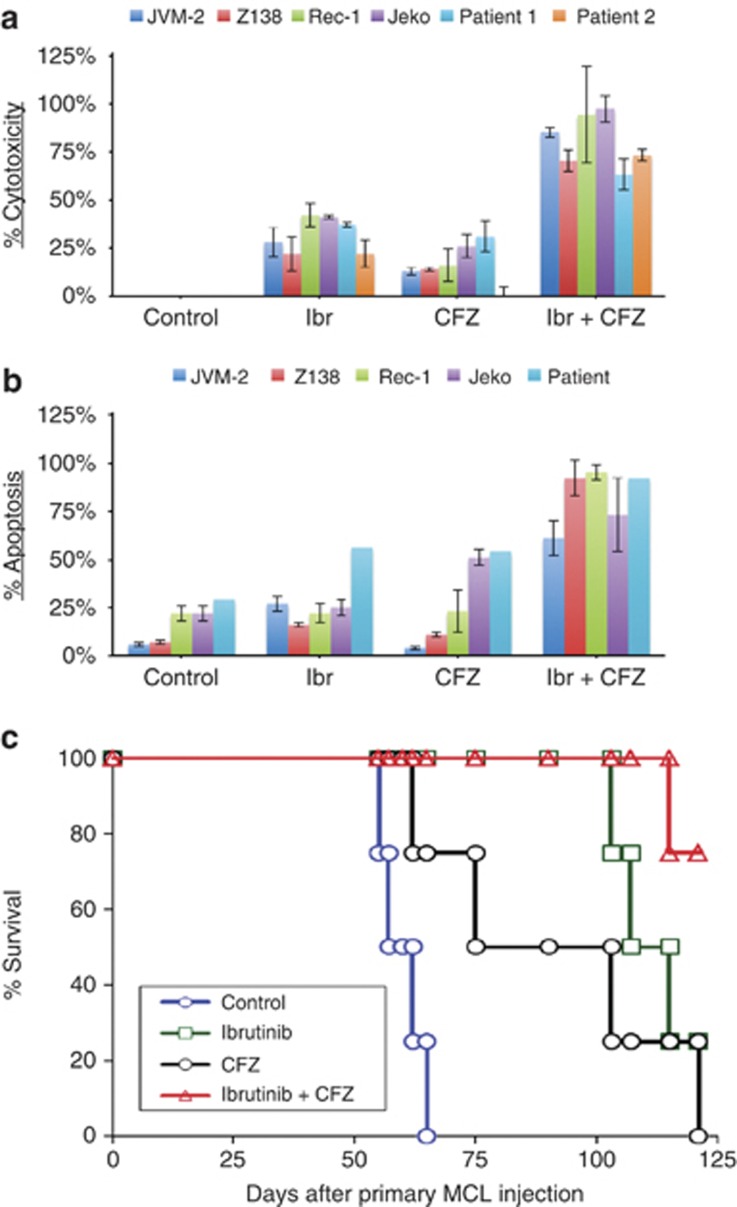
The combination of ibrutinib (Ibr) and carfilzomib results in synergistic cytotoxicity, apoptosis and enhances survival in MCL models. The cytotoxic and apoptotic effects of Ibr and carfilzomib as single agents and in combination were assessed in MCL cell lines and primary patient samples. Cells were treated for 48 h (Rec-1, Jeko and patient samples) or 72 h (JVM-2 and Z138) with the indicated drugs. Ibr concentrations ranged between 1.5 and 21 μM. Carfilzomib concentrations ranged between 2.6 and 20 nM. (**a**) Cytotoxicity was assayed using tetrazolium (MTS) (Rec-1, Jeko and patient samples) or alamarBlue (JVM-2 and Z138). (**b**) Apoptosis was assayed using Annexin V/propidium iodide staining (Rec-1, Jeko and patient samples) or cleaved Poly ADP Ribose Polymerase (PARP) staining (JVM-2 and Z138). (**c**) Primary patient MCL cells injected into human fetal bone chips, which had been subcutaneously implanted in SCID-hu mice. When human β_2_m was detectable in mouse serum, mice (five per group) were given Ibr 25 mg/kg, daily oral gavage and/or CFZ 5 mg/kg, intravenously twice a week for 5 weeks. Mice were killed once tumor burden reached 1.5 cm diameter (tumor burden equals mass diameter minus bone chip diameter in the long dimension). Kaplan–Meier survival curves of primary MCL-bearing SCID-hu mice were analyzed (Ibr plus CFZ versus Ibr/CFZ alone: *P*<0.01).

## Notes

MLW received research support from Pharmacyclics. MEW received research funding from Pharmacyclics and Janssen and is a consultant for Pharmacyclics. All other authors declare no conflict of interest.
