# Supplementary figures and images for: Combinatorial drug screening identifies synergistic co-targeting of Bruton's tyrosine kinase and the proteasome in mantle cell lymphoma
Source: Leukemia. 2013 Oct 8;28(2):407–10. doi: 10.1038/leu.2013.249 (PMC3918872; doi:10.1038/leu.2013.249)

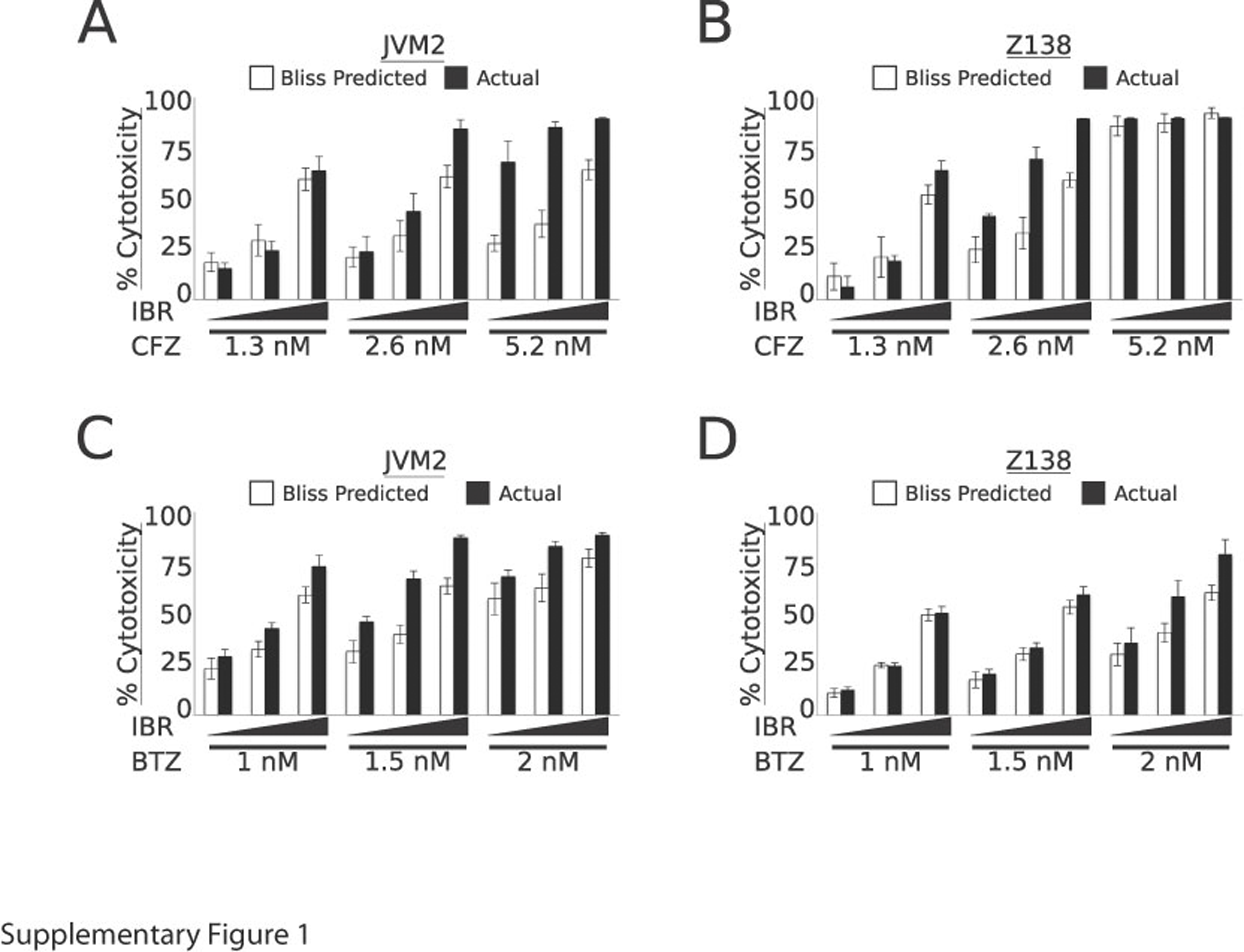

Supplement: Supplementary Figure 1 [file leu2013249x1.tif]

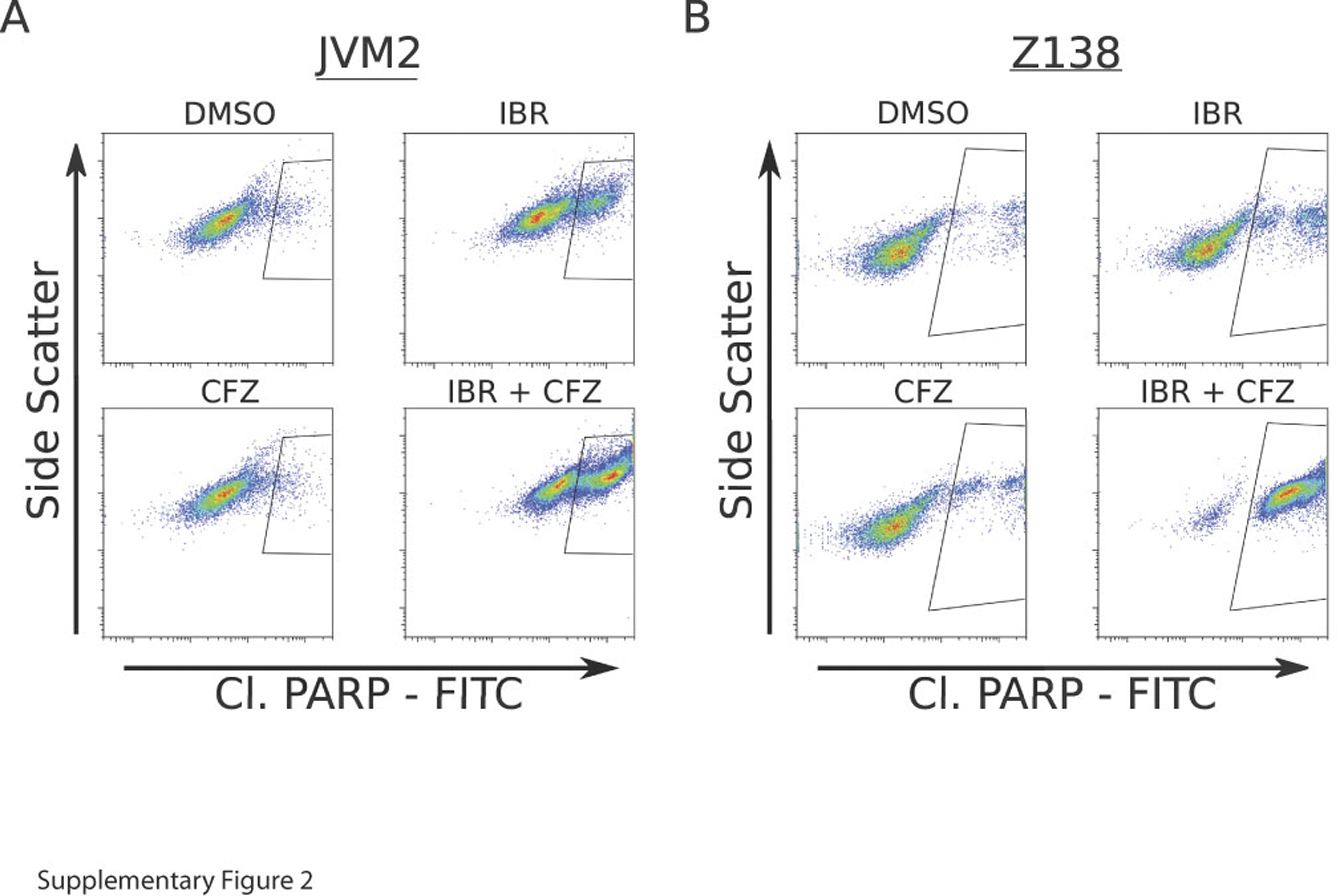

Supplement: Supplementary Figure 2 [file leu2013249x2.tif]
