# Supplementary material for: Combinatorial drug screening identifies synergistic co-targeting of Bruton's tyrosine kinase and the proteasome in mantle cell lymphoma
Source: Leukemia. 2013 Oct 8;28(2):407–10. doi: 10.1038/leu.2013.249 (PMC3918872; doi:10.1038/leu.2013.249)
Supplement: Supplementary Figure Legends [file leu2013249x3.doc]

**Supplementary Figure 1. The combination of ibrutinib and proteasome inhibitors results in synergistic cytotoxicity over a range of dose combinations.**

A-B. Ibrutinib (6 µM, 12 µM, 21 µM) was combined with carfilzomib (1.3 nM, 2.6 nM, 5.2 nM) in a 3x3 dose combination matrix in JVM-2 and Z138 cells. C-D. Ibrutinib (6 µM, 12 µM, 21 µM) was combined with bortezomib (1 nM, 1.5 nM, 2 nM) in a 3x3 dose combination matrix in JVM-2 and Z138 cells. White bars represent the average Bliss Predicted additivity value. Black bars represent the average actual cytotoxicity generated by the combination. Error bars represent the SEM. Each experiment was performed in biological triplicate.

**Supplementary Figure 2. The combination of ibrutinib and carfilzomib results in enhanced PARP cleavage.** JVM-2 (A) and Z138 (B) were treated as described in the text. Apoptosis was measured by flow cytometry assessing levels of cleaved PARP
