# Supplementary material for: Combinatorial drug screening identifies synergistic co-targeting of Bruton's tyrosine kinase and the proteasome in mantle cell lymphoma
Source: Leukemia. 2013 Oct 8;28(2):407–10. doi: 10.1038/leu.2013.249 (PMC3918872; doi:10.1038/leu.2013.249)
Supplement: Supplementary Table 1 [file leu2013249x4.doc]

Supplementary Table 1: Secondary Agents used for combinatorial screen

| **Drug** | **Target** | **Relationship to BCR pathway** | **Doses used** |
| --- | --- | --- | --- |
| Idelalisib (GS-1101, CAL-101) | PI3K | Proximal | 0.3125,0.625,1.25µM |
| Dasatinib | LYN | Proximal | 6.25,25,100µM |
| Enzastaurin | PKC | Proximal | 2.5,5,10µM |
| SC-514 | IKK | Proximal | 6.25,12.5,25µM |
| R-788 | SYK | Proximal | 0.6,1.35,2.85µM |
| Temsirolimus | mTOR | Proximal | 31.2,62.5,125nM |
| PD-0332991 | CDK | Distal | 125,250,500nM |
| PD-325901 | MEK | Distal | 6.25,12.5,25nM |
| Geldanamycin | Chaperone | Distal | 3.9,7.8,15.6nM |
| Panobinostat | HDAC | Distal | 1,2,4nM |
| Bortezomib | Proteasome | Distal | 1,1.5,2nM |
| Carfilzomib | Proteasome | Distal | 1.3,2.6,5.2nM |
| ABT-199 | BCL-2 | Distal | 0.75,1.25,2.5µM |
| Bendamustine | DNA replication | Distal | 3.125,6.25,12.5µM |
