# Supplementary material for: Combinatorial drug screening identifies synergistic co-targeting of Bruton's tyrosine kinase and the proteasome in mantle cell lymphoma
Source: Leukemia. 2013 Oct 8;28(2):407–10. doi: 10.1038/leu.2013.249 (PMC3918872; doi:10.1038/leu.2013.249)
Supplement: Supplementary Information [file leu2013249x5.doc]

**Supplementary Information: Materials and Methods**

Cell lines and Reagents:

JVM2, Z138, Jeko-1 and Rec-1 cells were obtained from ATCC. JVM2 cells were cultured in RPMI 1640 (Invitrogen, Carlsbad, CA, USA) with 10 mM HEPES (Invitrogen), 1 mM sodium pyruvate (Invitrogen), and 10% FBS (Gemini, Sacramento, CA, USA). Z138 cells were cultured in Iscove’s medium (Invitrogen) with 10% horse serum. Jeko-1 and Rec-1 cells were cultured in RPMI 1640 (Invitrogen) with 15% FBS (Invitrogen). All small molecule inhibitors for the initial screen were purchased from Selleck (Houston, TX, USA), with the exceptions of ABT199 (Active Biochemicals, Hong Kong, China), PD325901 (Pfizer, La Jolla, CA, USA), and SC-514 (EMD Biosciences). The cleaved PARP-FITC antibody and Annexin V / PI staining kit used for flow cytometry was from BD Biosciences (San Jose, CA, USA). MTS proliferation kit was purchased from Promega (Madison, WI, USA).

Human Sample Collection:

Specimens of peripheral blood or bone marrow aspirates were obtained from patients with MCL after obtaining informed consent. This project was approved by the Institutional Review Board at The University of Texas M. D. Anderson Cancer Center. Mononuclear cells were separated by Ficoll-Hypaque density centrifugation, and MCL cells were isolated using anti-CD19 magnetic microbeads (Miltenyi Biotec, Auburn, CA). The tumor cells were immediately treated *in vitro* or injected into SCID-hu mice.

Combinatorial Drug Screening:

Cells were plated at a density of 2x10^4^ cells/well (JVM2) or 1.5x10^4^ cells/well (Z138) in normal growth medium in 96 well plates. The following day, drugs were added to the plates. The cells were then incubated for 72 hours at 37^o^C and 5% CO_2_. AlamarBlue (Invitrogen) was then added to each plate, and the cells were incubated for an additional 4 hours. Relative cell number was then assessed using a fluorescence plate reader with a 540/25 nm excitation filter and a 620/40 nm emission filter. The data were analyzed by comparing the cytotoxicity caused by combination drug treatment to the predicted additive cytotoxicity calculated using the Bliss independence model.^12^

*In vivo* effects of ibrutinib plus CFZ in established MCL-bearing SCID mice:

Six-to 8-week-old male CB-17 SCID mice (Harlan, Indianapolis, IN, USA) were housed and monitored in the M.D. Anderson animal research facility. All experimental procedures and protocols were approved by the Institutional Animal Care and Use Committee of The University of Texas M. D. Anderson Cancer Center. Procedures for SCID-hu have been previously described.^15^ Approximately 4 to 6 weeks following implantation, one injection of 5 × 10^6^ purified patient MCL cells was administered directly into human fetal bone implants within SCID-hu hosts, after the mice were anesthetized with ketamine (75 mg/kg) - xylazine (12.5 mg/kg; Lloyd, Shenandoah, IA, USA). Mouse serum was collected, and the levels of circulating human β_2_Microglobulin (β_2_M) in mouse serum, obtained via a human β_2_M ELISA kit (Alpha Diagnostic, Owings Mill, MD, USA), were used to monitor tumor engraftment and growth in the SCID-hu mice. Once human β_2_M was detected in mouse serum, the mice received treatments with ibrutinib (25 mg/kg/day, oral gavage, daily), and/or CFZ (5 mg/kg/day, IV, twice a week for 5 weeks). Meanwhile, bone mass was measured lengthwise weekly after tumor cell inoculation. Mice were sacrificed once tumor burden reached 1.5cm diameter (tumor burden equals mass diameter minus bone chip diameter in the long dimension).

Flow Cytometric analyses:

Cleaved PARP: Cells were plated at a density of 1x10^6^ cells/well (JVM2) and 8x10^5^ cells/well (Z138) in 6 well plates. Cells were treated for 72 hours as described in the text, and then stained as previously described using cleaved PARP FITC antibody. *Annexin V/PI*: 1x10^5^ pretreated cells were washed with cold phosphate-buffered saline (PBS) and stained with an Annexin V^FITC^ and propidium iodide in accordance with the manufacture’s procedure (BD PharMingen, San Diego, CA, USA). Cells were assayed using a FACScantoll flow cytometer system (BD Biosciences) interfaced to FACS Diva software (v 6.0), and analyzed with Flow Jo (v7.2.1).
